# Impact of supervised drug consumption services on access to and engagement with care at a palliative and supportive care facility for people living with HIV/AIDS: a qualitative study

**DOI:** 10.7448/IAS.17.1.18855

**Published:** 2014-03-13

**Authors:** Ryan McNeil, Laura B Dilley, Manal Guirguis-Younger, Stephen W Hwang, Will Small

**Affiliations:** 1 British Columbia Centre or Excellence in HIV/AIDS, Vancouver, Canada; 2 Faculty of Health Sciences, Simon Fraser University, Burnaby, Canada; 3 Faculty of Education, Simon Fraser University, Burnaby, Canada; 4 Faculty of Human Sciences, Saint Paul University, Ottawa, Canada; 5 Centre for Inner City Health Research of the Keenan Research Centre of the Li Ka Shing Knowledge Institute, St. Michael's Hospital, Toronto, Canada

**Keywords:** HIV/AIDS, palliative care, supervised drug consumption services, harm reduction, highly active antiretroviral therapy, qualitative research

## Abstract

**Introduction:**

Improvements in the availability and effectiveness of highly active antiretroviral therapy (HAART) have prolonged the lives of people living with HIV/AIDS. However, mortality rates have remained high among populations that encounter barriers to accessing and adhering to HAART, notably people who use drugs. This population consequently has a high burden of illness and complex palliative and supportive care needs, but is often unable to access these services due to anti-drug policies and discrimination. In Vancouver, Canada, the Dr. Peter Centre (DPC), which operates a 24-bed residential HIV/AIDS care facility, has sought to improve access to palliative and supportive care services by adopting a comprehensive harm reduction strategy, including supervised injection services. We undertook this study to explore how the integration of comprehensive harm reduction services into this setting shapes access to and engagement with care.

**Methods:**

Qualitative interviews were conducted with 13 DPC residents between November 2010 and August 2011. Interviews made use of a semistructured interview guide which facilitated discussion regarding how the DPC Residence's model of care (a) shaped healthcare access, (b) influenced healthcare interactions and (c) impacted drug use practices and overall health. Interview transcripts were analysed thematically.

**Results:**

Participant accounts highlight how the harm reduction policy altered the structural-environmental context of healthcare services and thus mediated access to palliative and supportive care services. Furthermore, this approach fostered an atmosphere in which drug use could be discussed without the risk of punitive action, and thus increased openness between residents and staff. Finally, participants reported that the environmental supports provided by the DPC Residence decreased drug-related risks and improved health outcomes, including HAART adherence and survival.

**Conclusions:**

This study highlights how adopting comprehensive harm reduction services can serve to improve access and equity in palliative and supportive care for drug-using populations.

## Introduction

Advances in HIV treatment have improved survival among people living with HIV (PLHIV) [1, 2], but mortality rates remain high among populations facing social and structural barriers to accessing and adhering to highly active antiretroviral therapy (HAART) [3, 4]. PLHIV who use drugs are one population that commonly experiences suboptimal access to and utilization of HAART [5]. Poor HIV treatment outcomes among drug-using populations are driven by intersecting social determinants of health [6], including homelessness [7], stigma [8, 9] and drug law enforcement [10, 11]. Despite evidence suggesting that PLHIV who use drugs may benefit from HAART [12] and attain high levels of adherence [13], physicians in many jurisdictions do not initiate HAART with PLHIV who use drugs, with drug use and potential non-adherence often cited as justifications for denying treatment [14, 15]. Accordingly, PLHIV who use drugs suffer disproportionately from AIDS-related opportunistic illnesses [16] and premature mortality [17].

PLHIV who use drugs have a high level of need for palliative and supportive care – that is, care oriented towards preventing and relieving the suffering associated with life-threatening illnesses by addressing pain and other physical, psychosocial and spiritual care needs [18]. PLHIV frequently have untreated pain and a high prevalence of other symptoms, such as fatigue, anxiety, anorexia, nausea, vomiting and diarrhoea [19]. Comorbidities prevalent among drug-using populations, including hepatitis C (HCV) [20] and mental illness [21], may also need to be addressed and increase the complexity of care.

Although studies have shown that palliative and supportive care services improve outcomes among PLHIV, including reduced pain and anxiety [22], drug-using populations have poor access to these services [23, 24]. Palliative and supportive care services typically operate under abstinence-only drug use policies that serve as a structural-environmental barrier to care [23]. Accordingly, people who use drugs may not gain access to palliative and supportive care services, even when referred by a physician and supported by intensive case management [23]. Although research suggests that healthcare professionals affiliated with harm reduction services provide informal palliative care and support to drug-using populations [24], it appears that this population's palliative care needs go largely unmet.

Given ongoing barriers to palliative and supportive care services posed by abstinence-only drug policies [23], it has been argued that comprehensive harm reduction approaches, including supervised drug consumption services, should be integrated into these settings [23–25]. Removing the expectation of drug abstinence and promoting harm reduction practices has the potential to mediate access to care. We undertook this study to explore how the integration of comprehensive harm reduction services, including supervised drug consumption services, into a Canadian palliative and supportive care facility for PLHIV shapes access to and engagement with care.

### Study context

In the early 2000s, the Dr. Peter Centre (DPC), a healthcare facility in Vancouver (Canada) that includes a day health programme and residential palliative and supportive care programme for PLHIV, moved to integrate nurse-supervised injection services into its programming. The majority of DPC clients used illicit drugs [26], and changes to organizational policies were needed to engage this population and minimize drug-related harms. The DPC consulted with the College of Registered Nurses of British Columbia (CRNBC) and determined that the supervision of injections for the purposes of harm reduction was within the scope of registered nursing practice [27]. Furthermore, legal counsel advised that that this practice clarification meant that the organization held risk of civil liability if it did not permit nurses to supervise injections (e.g. liability for injection-related injuries), while having only limited risk of criminal prosecution. The DPC integrated supervised injection services into its model of care in 2002 and later revised its harm reduction policy to accommodate the smoking of illicit drugs in an outside area. The DPC has since been issued a letter in support of its harm reduction approach by the Vancouver Coastal Health Authority, which is responsible for licensing residential care facilities in this region.

The DPC's harm reduction approach aims to minimize drug-related harm and mediate access to clinical and support services for PLHIV who use drugs. Although the supervised injection room integrated into the Day Health Program has been previously examined, and has been found to encourage safer injecting practices and improve access to nursing care [28], the DPC Residence has yet to be studied despite its status as the only residential healthcare programme in North America in which open drug use occurs. In this 24-bed palliative and supportive setting (residents each have their own room), residents may use drugs and have a range of harm reduction supports available to them (see Table 1), with nurses supervising injections when requested. The DPC Residence also provides health and environmental supports, including but not limited to 24-hour specialized nursing care, HAART administration, pain and symptom management, nutrient-dense meals and counselling.

**Table 1:** Supports provided by the DPC Residence

The harm reduction approach taken by the DPC Residence seeks to mediate access to palliative and supportive care services, and minimize drug- related harm. DPC residents have been disproportionately affected by intersecting social and structural determinants of health, such as homelessness and food insecurity, and typically have complex comorbidities (e.g. mental illness and hepatitis C) in addition to HIV/AIDS.
**Harm reduction supports available to residents include:**
*1. Access to harm reduction supplies*
• Harm reduction supplies (e.g. syringes, disposable cookers, tourniquets, alcohol swabs, Pyrex stems, mouthpieces and screens) may be accessed 24 hours a day, 7 days a week.
• Harm reduction supplies are located on a cart located in a discreet location and may also be requested from nurses.
*2. Nursing support*
• Nurses supervise injections in residents’ rooms as requested.
• Nurses are not permitted to administer injections but provide safer injecting education, which includes *in situ* demonstrations of safer injecting techniques.
• Nurses are trained to administer naloxone and directed to contact the emergency medical services located at the hospital adjacent to the DPC Residence in the event that a resident experiences an overdose.
• Nurses are trained to manage residents who experience stimulant over-amping and closely monitor residents for symptoms of stimulant toxicity. Nurses are directed to immediately contact emergency medical services at the nearby hospital in the event that the resident experiences stimulant toxicity or acute mental distress.
• Nurses are not permitted to possess drugs and are required to contact police if drugs come into their possession so that they may be safely disposed of.
• Nurses dispense methadone to residents enrolled in methadone treatment.
*3. Environmental support*
• Resident rooms are treated as private residences (residents receive social assistance payments, and a portion of this income is deducted as rent), and residents may inject in their rooms without supervision.
• Resident rooms are equipped with emergency pull cords and sharps containers. Although residents are not permitted to smoke drugs in their rooms, they may do so in an outside area.

## Methods

This study is based upon qualitative interviews conducted with DPC residents between November 2010 and August 2011. We chose to conduct qualitative interviews to generate insights into how the harm reduction supports are experienced by residents [29]. Ethical approval was obtained from the research ethics boards at the University of British Columbia and Saint Paul University. We also obtained written permission from the DPC to access the residence.

All DPC residents were eligible to participate in this study. We sought to interview at least half of the 24 residents to ensure that our data captured a broad range of participant experiences. Several participants (*n*=3) were recruited through research advertisements posted in the DPC Residence. All remaining participants (*n*=10) were recruited through referral from a DPC staff member, who used an approved script and assent protocol to obtain permission to release their contact information to our research team. All DPC residents were approached by the staff member regarding potential participation in our study, and 11 individuals agreed to have their contact information released to our research team. These 11 individuals were visited at the DPC Residence by the lead author, who provided them with further information regarding the study. Ten of these individuals agreed to participate in an interview.

The lead author interviewed a total of 13 participants in their rooms at their convenience. Written informed consent was obtained prior to the interview. An interview topic guide was used to facilitate discussion and addressed a range of topics, including how the DPC Residence's harm reduction supports (a) shaped healthcare access, (b) influenced healthcare interactions and (c) impacted drug use practices and overall health. Participants each received a $20 CAD honorarium for participating in this study. Interviews were recorded and lasted 30–90 minutes, although most were approximately 45 minutes in length. Interviews were transcribed verbatim by a research assistant and reviewed by the lead author for accuracy.

Our analysis focused on how the harm reduction supports provided by the DPC Residence shaped access to and engagement with palliative and supportive care services. Interview transcripts were imported into NVivo qualitative analysis software [30] to assist with data management and coding. We used a coding framework made up of *a priori* categories extracted from the interview topic guide to facilitate analysis. Data were coded independently by two team members (RM & LD), who met regularly with the larger team to discuss emerging themes and revise the coding framework. Once the final categories were established, the lead author recoded the data to enhance their validity.

## Results

### Sample characteristics

Thirteen individuals participated in this study (10 men and three women). The average age was 48 years old (range: 36–62 years). Six participants self-identified as members of a visible minority, with three reporting Aboriginal ancestry. All participants reported a history of illicit drug use, and 10 reported illicit drug use within the past 30 days, with crystal methamphetamine (*n*=6), crack cocaine (*n*=5) and powdered cocaine (*n*=3) reported as the most commonly used drugs. Four participants reported polysubstance use within the past 30 days.

### Mediating access to palliative and supportive care

Participants described how the harm reduction supports mediated access to palliative and supportive care services by contrasting their experiences at the DPC Residence with those in hospital settings. Many participants reported frequent hospitalizations prior to admission to the DPC Residence, and reported that they generally left hospital against medical advice. Their decisions to leave hospital against medical advice were the consequence of inadequate pain and withdrawal management and policies prohibiting drug use. Several participants reported that they routinely left hospital to alleviate symptoms associated with opiate withdrawal or due to the craving associated with active addiction. For example:

In [the hospital], I had pneumonia or something. I had this abscess on my side … they had to lance that. Once I got to the point that I didn't need my IV, I knew it was welfare day and I had my cheque. I know that I could go and get it and cash it. So, I got like 600 bucks in my pocket and I decided, “Fuck this! [I'm] fucking out of here to go get wired.” [Participant #4, Male]

Several participants left hospital due to unmet pain management needs, especially if they believed that, in the words of one participant, “doctors used medication as a disciplining measure … lowering or raising the dosage according to how you were behaving” [Participant #10, Male]. Many participants emphasized that leaving hospital against medical advice adversely impacted HIV and other health outcomes, which led to their referral to the DPC Residence.

By providing harm reduction supports and accommodating onsite drug use, the DPC Residence altered the structural-environmental context of healthcare services delivery. Participants reported that these harm reduction supports mediated access to palliative and supportive care, and made them feel *welcome*. Many participants reported that they would have declined referrals to the DPC Residence had these harm reduction supports not been in place. For example:

You get to inject the drugs you want to inject. If you want to inject cocaine, you can. If you want to inject heroin, you can. I wouldn't be able to live here [if these harm reduction supports were not available]. I would just go nuts if I couldn't inject. I get sick, you know. I get really dopesick. [Participant #13, Female]

### Reshaping healthcare interactions

Participant accounts highlighted how the harm reduction approach fostered an atmosphere in which drug use could be discussed without the risk of punitive action (e.g. being *kicked out*). Participants contrasted their relationships with DPC staff with those with hospital staff, noting that the latter were characterized by mistrust due to the enforcement of abstinence-only drug policies. Many participants reported that they limited their interactions with hospital staff to avoid being caught using or in the possession of drugs. As one participant explained, “In the hospital, you look over your shoulder and hope to not get caught. They embarrass you” [Participant #5, Female].

In contrast, most participants expressed that they were comfortable discussing their drug use and, in turn, health needs with DPC staff. This increased openness with DPC staff improved healthcare interactions and led to the development of trusting relationships. As one participant noted, “You don't have any hesitations [when approaching nursing staff]. If something pops up, I don't hesitate to ask any questions” [Participant #10, Male]. A minority of participants reported that they did not discuss their drug use with DPC staff because they believed it was “private” [Participant #8, Male] or “didn't want to inflict it on anyone else” [Participant #11, Male]. However, these participants reported that they were comfortable approaching DPC staff regarding health needs.

Furthermore, participants articulated how the model of care adopted by the DPC Residence, which acknowledges that drug-related harm is driven by social and structural inequities, minimized drug-related stigma. Participants emphasized that DPC staff did not discriminate against them on the basis of their drug use. For example:

At least they know why [I use drugs]. They know how my lifestyle was before and it is the understanding part that I like. They say they are not here to judge us by what we do but by how they take care of us. [Participant #6, Female]

### Enabling safer drug use practices

Participant accounts illustrated how healthcare (i.e. harm reduction supplies and nurses) and environmental supports (i.e. clean and regulated environment) were critical to minimizing drug-related harm. Importantly, many participants reported that they were sometimes unable to access harm reduction supplies prior to admission to the DPC Residence and reused or borrowed syringes or pipes. The DPC Residence minimized these risk behaviours by ensuring that participants were able to access harm reduction supplies at all times via a harm reduction cart located in discreet locations. As one participant noted:

If I need [injecting equipment], it's right here …. You don't have to go and grab a dirty [syringe] like when I was on the street. For a while, I was down on Hastings [i.e. living on the streets in the street-based drug scene] and that is what I'd do. [Participant #4, Male]

Participants expressed that the DPC Residence was “cleaner” than unregulated drug use settings (e.g. alleys and public washrooms), and thus reduced their susceptibility to opportunistic infections. One participant with a history of injection-related infections noted, “It's cleaner and safer. It's better than injecting in an alley or behind a dumpster” [Participant #8, Male].

Approximately half of the participants who injected drugs reported that they regularly asked nurses to supervise injections, which they identified as *safer* than injecting alone. These participants reported that nurse-supervised injection services reduced the likelihood of drug-related mortality because nurses were trained to respond to overdoses and stimulant toxicity. As one participant noted:

I think it's better to do something where you're safe … I know, if I'm gonna get in trouble [i.e. overdose], I am going to be looked after. [Participant #7, Male]

However, several participants continued to inject alone, with several only asking nurses to supervise injections when they were injecting drugs from an unfamiliar source. These participants believed that they did not require supervision because they had learned to minimize overdose and other drug-related risks over the course of their injecting careers. As one participant stated, “I'm not stupid with my drugs anymore. I have to taste it and see if it is real. I'll do a taste of it or just smoke it” [Participant #4, Male].

### Improving health and quality of life

Participants expressed that the DPC Residence mitigated the impact of social and structural determinants of health (e.g. homelessness and food insecurity) by offering a range of health and environmental supports. Importantly, all participants reported that they were able to adhere to treatment, including HAART regimens, because medications were administered by nurses in a structured environment. For example:

I can actually take them [HAART medications] at the times that I am supposed to. When I was on the street, I would miss a couple of days. It was a waste of time. [Participant #5, Male]

Furthermore, participants emphasized the role of broader environmental supports, such as housing and nutritional services, in improving overall health. All participants reported that the availability of these supports, in combination with increased HAART adherence and regular nursing care, had improved their HIV outcomes. As one participant noted:

My CD4 count was below 20 [prior to admission]. Now, I got it at 180 …. So, that means I'm doing a lot better. I'm 185 pounds, so I've probably gained 30 pounds in the last three months …. I saw the doctor yesterday and he said, “Slow down! Start working out!”. [Participant #2, Male]

Accordingly, the majority of our participants reported that they had lived far longer than their healthcare providers had predicted, which they largely attributed to the care and support provided by the DPC Residence. Several participants reported that these improvements in their health allowed them to be discharged to supportive housing programmes that operate in connection with the DPC Residence. These participants continued to access health and social care services (e.g. nursing care, medication support and nutritional services) provided as part of the DPC's Day Health Program, as well as other community health agencies. However, because the complexity of their healthcare needs (which included comorbid mental illness) led to suboptimal HAART adherence and deteriorations in their overall health, these participants required a level of care that led to their eventual readmission to the DPC Residence. As one of these participants noted, “I came here to die but, every time I come here and have a life threatening illness, they save me” [Participant #5, Female].

## Discussion

In summary, we found that the harm reduction approach taken by the DPC Residence altered the structural-environmental context of healthcare services, and thereby increased access to and engagement with palliative and supportive care. We found that the harm reduction approach was critical in (a) fostering an environment in which PLHIV who use drugs felt welcome, (b) improving healthcare interactions by minimizing drug-related stigma and (c) enabling safer drug use practices by promoting harm reduction. Furthermore, increased access to healthcare and environmental supports, including HAART, resulted in improved HIV treatment outcomes and survival.

Growing awareness that abstinence-based drug policies constrain access to inpatient health services among drug-using populations has sparked calls to integrate harm reduction approaches into these settings [25, 31]. To date, existing drug laws have largely prevented the scaling up of harm reduction approaches in these settings, and thus limited evaluations of models of harm reduction healthcare. Although our study was conducted in a palliative and supportive care setting, it generates preliminary insights into the potential role of harm reduction approaches to minimize barriers to inpatient healthcare services.

Our findings illustrate how changes to the structural-environmental context of healthcare services delivery (i.e. accommodating illicit drug use in accordance with a harm reduction approach) improve access to and engagement with care among PLHIV who use drugs. In this regard, our findings underscore the importance of offering HIV care services tailored to the need of PLHIV who use drugs. Further studies are needed to determine whether this approach improves access to care at the population level and in other healthcare settings (e.g. hospitals). This is especially urgent in settings where PLHIV who use drugs experience disproportionately high mortality levels and encounter difficulties accessing care or completing treatment.

However, given continued opposition to harm reduction programming and the emphasis on drug law enforcement in many settings, efforts to integrate supervised drug consumption services into health settings are likely to encounter opposition. In many jurisdictions, anti-drug laws may restrict the expansion of harm reduction services and potentially lead to legal liabilities for health and HIV care organizations implementing these approaches. It may be the case that scaling up harm reduction approaches is only possible in settings where existing drug laws are flexible enough to permit the expansion of these services, or where urgent action is needed to respond to HIV epidemics among local drug-using populations. It is worth noting that although the DPC lacks an exemption to federal drug laws, it enjoys the support of key stakeholders (e.g. the local health authority and CRNBC) because of the acknowledgement that innovative public health strategies were needed to respond to an HIV epidemic within the local drug-using population.

Our findings illustrate how accommodating illicit drug use within health settings and reorienting organizational policies to emphasize harm reduction have the potential to improve the quality of care, while reducing drug-related harms. Consistent with research in other harm reduction healthcare settings [28, 32–34], participants emphasized how the approach taken by the DPC Residence minimized stigma, and fostered an atmosphere in which they could engage with healthcare staff without the fear of punitive action. Given that experiences of drug-related stigma in healthcare settings are a widely acknowledged barrier to care for drug-using populations [35], this finding has particular salience.

Our findings also illustrate how increased access to harm reduction supplies and environmental supports reduced drug-related risks. Consistent with previous research on supervised injection facilities [36, 37], the DPC Residence enabled participants to consume drugs in a safe, regulated environment and adhere to harm reduction practices. Participants believed that the DPC Residence reduced mortality risks when injecting opiates or stimulants because they could inject in accordance with harm reduction practices and nurses were available to supervise injections and respond to overdoses or stimulant toxicity. Participants also reported that the DPC Residence reduced vulnerability to injection-related infections, which disproportionately impact PLHIV [38]. These reductions in drug-related harm may help to allay fears regarding the potential adverse impacts of the harm reduction model in healthcare settings, especially given that these complications would likely recur if individuals were required to leave the premises to consume drugs.

Finally, our findings build upon increased calls for the integration of palliative and treatment approaches to HIV/AIDS care – that is, to provide palliation while also administering HAART [19, 39, 40]. While it has been argued that combining these approaches may improve HIV outcomes among underserved populations, limited research has been undertaken exploring models of care blending these approaches. While larger-scale studies are needed to determine the impact of this approach at the population level, it is noteworthy that participants reported that being administered HAART in a welcoming and structured environment increased adherence, and subsequently improved survival and quality of life. Our findings suggest that integrating palliative and treatment approaches may require the development of services, such as the DPC Residence, that emphasize chronic disease management alongside more traditional palliative and supportive care, while also tailoring their services to the needs of underserved populations.

This study has several limitations. Although a small sample size is common among studies in palliative and supportive care settings, our study relies on a small sample size that may not be representative of the experiences of all PLHIV who use drugs and are in need of palliative and supportive care. Our findings may have limited transferability to settings where healthcare is organized differently. Finally, although participants reported improvements in health, including HAART adherence, further research is needed to fully identify the impact of this programme on these outcomes.

## Conclusions

Our study generated insights regarding the integration of comprehensive harm reduction services into a residential palliative and supportive care setting. This approach was found to mediate access to care for PLHIV who use drugs, while producing a range of positive outcomes (e.g. reduced drug-related harms and improved healthcare interactions). This approach resulted in improved access to and utilization of HAART, and thus improved survival. Those concerned with increasing access and equity to care for PLHIV who use drugs, including HAART adherence, would be well served to consider this approach.
